# Partial sleep in the context of augmentation of brain function

**DOI:** 10.3389/fnsys.2014.00075

**Published:** 2014-05-01

**Authors:** Ivan N. Pigarev, Marina L. Pigareva

**Affiliations:** ^1^Institute for Information Transmission Problems (Kharkevich Institute), Russian Academy of SciencesMoscow, Russia; ^2^Institute of Higher Nervous Activity and Neurophysiology, Russian Academy of SciencesMoscow, Russia

**Keywords:** local sleep, cerebral cortex, slow wave sleep, sleep function, visceral control

## Abstract

Inability to solve complex problems or errors in decision making is often attributed to poor brain processing, and raises the issue of brain augmentation. Investigation of neuronal activity in the cerebral cortex in the sleep-wake cycle offers insights into the mechanisms underlying the reduction in mental abilities for complex problem solving. Some cortical areas may transit into a sleep state while an organism is still awake. Such local sleep would reduce behavioral ability in the tasks for which the sleeping areas are crucial. The studies of this phenomenon have indicated that local sleep develops in high order cortical areas. This is why complex problem solving is mostly affected by local sleep, and prevention of local sleep might be a potential way of augmentation of brain function. For this approach to brain augmentation not to entail negative consequences for the organism, it is necessary to understand the functional role of sleep. Our studies have given an unexpected answer to this question. It was shown that cortical areas that process signals from extero- and proprioreceptors during wakefulness, switch to the processing of interoceptive information during sleep. It became clear that during sleep all “computational power” of the brain is directed to the restoration of the vital functions of internal organs. These results explain the logic behind the initiation of total and local sleep. Indeed, a mismatch between the current parameters of any visceral system and the genetically determined normal range would provide the feeling of tiredness, or sleep pressure. If an environmental situation allows falling asleep, the organism would transit to a normal total sleep in all cortical areas. However, if it is impossible to go to sleep immediately, partial sleep may develop in some cortical areas in the still behaviorally awake organism. This local sleep may reduce both the “intellectual power” and the restorative function of sleep for visceral organs.

## Sleep and “human factor”

The modern industrial style of life and necessity to live in non-natural environments often leads to dramatic errors in human behavior. Usually these errors are included in the general term “human factor” and are attributed to reduced attention, inability to predict consequences of an action in complex conditions and, finally, to reduced brain ability. Thus, the intention to augment the brain function can be considered a challenging goal for Neuroscience.

It was noticed that industrial anthropogenic disasters, traffic accidents, and medical errors often occur during the night, suggesting a probable link between sleepiness and reduced quality of brain functioning (Mitler et al., 1988; Dinges, 1995; Dinges et al., 1997; Cajochen et al., 1999; Barger et al., 2006; Akerstedt et al., 2011). Given the importance of this issue, many special studies devoted to investigation of the possible link between sleep disturbances and mental abilities were undertaken.

It was demonstrated that short-term total sleep deprivation results in cognitive impairments, especially in learning, and memory tasks (Maquet, 2001; Stickgold, 2005; Born et al., 2006; Walker, 2008, 2009; Diekelmann and Born, 2010; McCoyand and Strecker, 2011; Diekelmann et al., 2012, 2013).

In some studies, authors concluded that periods of slow wave sleep (SWS) played a particular role in these processes (Fowler et al., 1973; Plihal and Born, 1997; Diekelmann et al., 2011). Others maintained a rapid eye movement (REM) sleep dependency (Empson and Clarke, 1970), although in some studies REM sleep deprivation had no effect on different aspects of memory function (Hornung et al., 2007; Saxvig et al., 2008).

In addition to the function of memory consolidation, sleep has been proposed to benefit the encoding of new information during succeeding periods of wakefulness (McDermott et al., 2003; Yoo et al., 2007; Mander et al., 2011).

## Peculiarities of sleep deprivation effects

The impact of sleep deprivation on memory formation did not appear to be universal, but instead, differed on the basis of many factors such as the types of tasks used for learning, and personal characteristics of subjects, including their emotionality. It has been shown that sleep-dependent motor skill memory improvement was dependent on the nature of the skill to be learned (Cohen et al., 2005; Cohen and Robertson, 2007; Siengsukon and Boyd, 2008). In another study, perception, attention and memory were impaired by sleep deprivation, but visual search and logical reasoning tasks were not (Williamson et al., 2001). A deficit in perceptual classification ability in an information-integration task was observed for some, but not all, sleep-deprived individuals (Maddox et al., 2009).

In their review of the sleep deprivation literature, Harrison and Horne (2000) concluded that sleep deprivation has little effect on simple rule-following tasks, but it obstructs decision making in complex integration tasks requiring flexibility, innovation or plan revision. However, other researchers observed performance decrements in relatively simple tasks such as identification and vigilance tasks (Chee et al., 2008; Ratcliff and Van Dongen, 2009). Sleep deprived subjects exhibited decreased performance in the taking advantage task (Glass et al., 2011).

Genzel et al. (2009) used different deprivation conditions throughout the experiment and did not find that an intense decrease in the total amount of REM sleep or SWS of their volunteers led to the inhibition of learning. They even proposed that sleep-dependent memory consolidation did not rely only on intact amounts of SWS or REM sleep across a night, but required different EEG microstructures, e.g., sleep spindles, δ-waves, and PGO waves.

It was proposed that reduced visual short-term memory after sleep deprivation may be connected, not with impairment of memory consolidation mechanisms but, rather, with a decline in visual attention and/or visual processing (Chuah and Chee, 2008).

An important factor was the length of time between training and test or amount of skill practice, independent of whether there was sleep or not (Shadmehr and Brashers-Krug, 1997; Robertson et al., 2004; Keisler et al., 2007; Song et al., 2007; Criscimagna-Hemminger and Shadmehr, 2008; Doyon et al., 2009; Debas et al., 2010; Borich et al., 2011; Borich and Kimberley, 2011; Voderholzer et al., 2011; Reis et al., 2013). In a nap study (Mander et al., 2011) no differences were observed between the sleep and no sleep groups in a specific alertness control task.

Results of sleep deprivation experiments were often rather contradictory. According to Lo et al. (2012), influence of sleep deprivation depends on the task domain, prior sleep debt, circadian phase at which performance is assessed, and genetically determined subject characteristics. Degree of task difficulty and the subject’s emotionality influence the outcome of experiments concerning the connection between sleep and memory (Smith, 2001; Walker and Stickgold, 2006). Small differences in test design can cause large discrepancies in the studies of sleep dependency of memory processes. This may explain why some results are regularly found only by the same groups of scientists (Genzel et al., 2009).

Even in those experiments where positive effects of sleep on memory consolidation were demonstrated, these effects were very small. It seemed unlikely to us that the only function of sleep was simply to provide such a modest improvement in memory. On the other hand, the probable connection of sleepiness with the rare but dramatic consequences of anthropogenic disasters, as well as the fantastic pictures of dreams, support the general belief that the first function of sleep is for efficient functioning of the brain.

## Modern theories of sleep

Several modern theories concerning the function of sleep offer hypothetical mechanisms, which could be used by the brain for this purpose, e.g., the theory of neuronal groups (Krueger and Obál, 1993; Krueger et al., 2008) or the theory of synaptic homeostasis (Tononi and Cirelli, 2003, 2006). All theories, which were based on the assumption that sleep first of all is important for efficient brain function referred to numerous studies which demonstrated, both in humans and animals, “local use dependent processes”. It was shown that δ-power during the first hours of sleep is higher in those cortical areas, which were more active immediately before sleep (Kattler et al., 1994; Rector et al., 2005; Huber et al., 2006). δ-power was considered therefore as an indicator and measure of recuperative processes in the brain.

However, if to accept an idea that function of sleep is to keep the efficient brain function it would be logical to expect that the brain would be the organ most vulnerable to sleep deprivation. However, the results of fundamental studies of A. Rechtshaffen and his colleagues (Everson et al., 1989; Cirelli et al., 1999; Rechtshaffen and Bergmann, 2002) do not confirm this suggestion. Their experiments demonstrated that total sleep deprivation led first of all to multiple visceral disorders (hair loss, skin and gastro-intestinal ulcerations and so on) and, finally, to unavoidable death of animals. A striking finding was that in rats that died after several days of total sleep deprivation, the only organ, which did not have any obvious degenerative changes, was the brain (Cirelli et al., 1999). This observation is surprising, since the negative effects of sleep deprivation on mental ability are well known. However, investigation of neuronal activity in the cerebral cortex in the sleep-wake cycle offers insights into the other sleep dependent mechanisms which can explain the reduction of mental abilities for complex problem solving, even in the normally working brain.

## Phenomenon of local (partial) sleep

It was generally assumed for a long time that sleep develops synchronously in all areas of the mammalian cortex. The only exception has been reported for dolphins, whose EEG show periods of deep SWS in either the right or left hemisphere alone. Such periods of unilateral sleep may last for more than 2 h. Even in dolphins, however, EEG activity in different areas of one hemisphere was always found to be synchronized, or desynchronized simultaneously (Mukhametov et al., 1977; Mukhametov, 1984).

However, later it was shown that in terrestrial animals, in particular conditions, sleep developed only in some cortical areas during behavioral wakefulness. Thus, such partial sleep might be especially dangerous, because neither the person himself nor other individuals could notice its appearance and development. At the same time, dangerous consequences of temporal disengagement of some cortical areas from the control of behavior potentially can be rather dramatic. That is why understanding the physiological mechanisms involved in initiation of sleep, and particularly of local sleep, can be considered an important element in attempts to augment brain functionality.

The phenomenon, which later was called “local sleep” was described in a study of the cat’s frontal eye field (Pigarev, 1984). Neurons in this frontal cortical area strongly responded to visual stimulation only during periods of high behavioral alertness, and often became visually unresponsive during subsequent periods of quiet wakefulness. However, any sensory stimulation raising alertness (visual, auditory or olfactory), restored visual responses in this cortical area.

More detailed description of such unusual neuronal behavior in the cerebral cortex, and the term “local or partial sleep” appeared in the investigation of cortical visual area V4 in experiments with behaviorally awake monkeys (Pigarev et al., 1996, 1997; Pigarev, 1997). It was demonstrated in long-running experiments, that when a monkey had to perform a monotonic visual discrimination task, neurons became less responsive to the same visual stimuli and finally stopped responding at all, while the monkey continued to work in the task. If the task was interrupted, the monkey fell asleep for 10–20 min. After the nap, neuronal responses to visual stimuli often recovered. Neuronal background firing during such periods of temporal inactivity resembled that which these neurons demonstrated during periods of natural sleep of the animal. The monkey’s performance in the visual task during periods of local sleep in the area V4 was rather high, although it was slightly reduced in comparison with that at the beginning of the experiment. Thus, one could conclude that at least visual area V1 still was working. In the same study it was noticed that, even within the area V4, local sleep developed not simultaneously but started from the periphery of the visual field. The last neurons, which were recruited in sleep, were neurons in the region of the foveal representation.

It was obvious that spread of sleep started from the “higher order” sensory areas most likely crucial for the most complicated behavioral situations. Taking all those considerations into account, one could expect to observe local sleep more often in organisms with better expressed multiple sensory representations, and most of all in primates. Indeed, the fronto-occipital trend in δ-power most pronounced during beginning of sleep was discovered in human subjects by the group of A. Borbély (Werth et al., 1996, 1997). Extensive studies of sleep spread dynamics are presented and discussed in the review of Ferrara and De Gennaro (2011).

We would like to remind here that since 1993 (Krueger and Obál, 1993; Kattler et al., 1994) the mentioned above idea concerning local use dependent sleep was widely discussed in sleep literature. Although experiments demonstrated local use dependent sleep, and observations of local sleep in behaviorally awake animals were mutually supportive, they were not absolutely identical. In the first case it was shown that in sleeping brain the depth of sleep can locally vary from region to region dependent on the previous history of activation. In the second case sleep in some cortical areas appeared during behavioral wakefulness. Namely this second case we will have in mind using the term “local sleep” in future.

Unexpected results obtained by Drummond et al. (2001, 2004) most likely were also connected with dynamics of local sleep spread over the human cerebral cortex. Investigating the effects of 35 h total sleep deprivation on memory impairments in verbal learning tasks using fMRI, these researchers demonstrated increased activity in frontal and parietal cortical regions, absent in the control non-deprived group. This can reflect partial sleep development in these high order associative cortical areas caused by sleep deprivation.

It seemed less likely that local sleep would be found in animals with more simple cortical organization and a limited number of sensory areas. However, recently, local sleep was described in behaviorally active rats, and again in the frontal cortical area (Vyazovskiy et al., 2011).

## Local sleep and cognitive impairments after sleep deprivation

Taking into account the phenomenon of local sleep, the cognitive impairments after sleep deprivation can be explained not by the general deterioration of brain efficiency, but by switching of several cortical areas from those functions, which they have to perform in wakefulness.

At first sight, the hypothesis that local sleep is responsible for a reduction of brain functionality looks similar to the proposal that after a long period of wakefulness, the brain needs recuperation during sleep. The difference is only in the temporal sequence of events. After prolonged wakefulness, all regions of the brain may fall asleep for recovery simultaneously, or certain areas may do so at first, while others remain awake for some time.

However, it is possible to consider another, fundamentally different, scenario. What if the brain, as all other visceral organs of an organism, does not need any special recuperative rest connected with total interruption of functionality? What if the brain, like a computer, can work efficiently for long periods of time, and observed “sensory isolation” of the brain during sleep just reflects switching over for processing of another flow of incoming information?

We should not forget that sleep-deprived animals die not because they become blind, deaf, have forgotten the ways to a food tray or because of serious problems with decision making. They die mainly because of multiple visceral disorders in virtually all life supporting systems, including the immune system (Rechtshaffen and Bergmann, 2002). At the same time, the brain appears to be the most resistant organ.

Our observations of the neuronal activity in the sleep-wake cycle also did not convince us that during long periods of wakefulness there were crucial pathological changes of the neuronal state, which forced a brain to switch into a sleeping “restorative” mode. Nevertheless, one can argue that it is generally recognized that the pattern of SWS EEG is very specific for this state, and differs from the pattern of EEG in wakefulness. However, interpretation of this observation also is equivocal. The EEG pattern of active SWS was usually compared with the EEG pattern in a state of very passive wakefulness, when human subjects or animals were immobile and without intensive sensory stimulation.

## Whether cortical EEG reflects peculiarity of brain activity in wakefulness and sleep, or just pattern of the cortical afferent flow?

**Figure 1:**
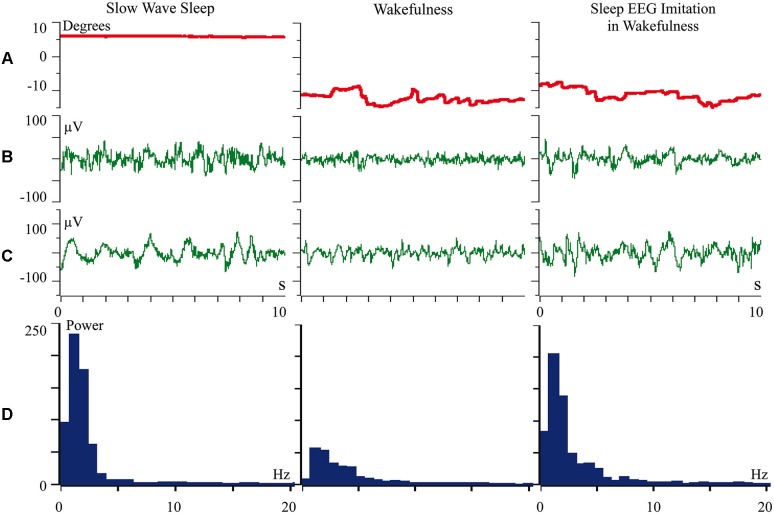
**Rhythmic stimulation by the optimal visual stimulus during wakefulness evokes sleep like slow wave activity in the cat visual cortex**. The rows: **(A)** vertical component of eye movements which helps to distinguish sleep (upward deviation) from wakefulness (downward deviation); **(B)** general EEG recorded between electrodes over temporal and frontal cortical areas of the cat; **(C)** local field potentials (local EEG) recorded between two tungsten microelectrodes located 300 μm one from another within the cortical visual area V1; **(D)** power spectrum of the local EEG presented above in **panel C**. All parameters were collected during SWS (left column), during passive wakefulness (middle column), and during the procedure of “sleep EEG imitation” by visual stimulation in wakefulness, which produced strong excitation of the cortical neurons recorded by the microelectrodes (right column). Technical details of the study in Pigarev et al. (2013).

**Figure 2:**
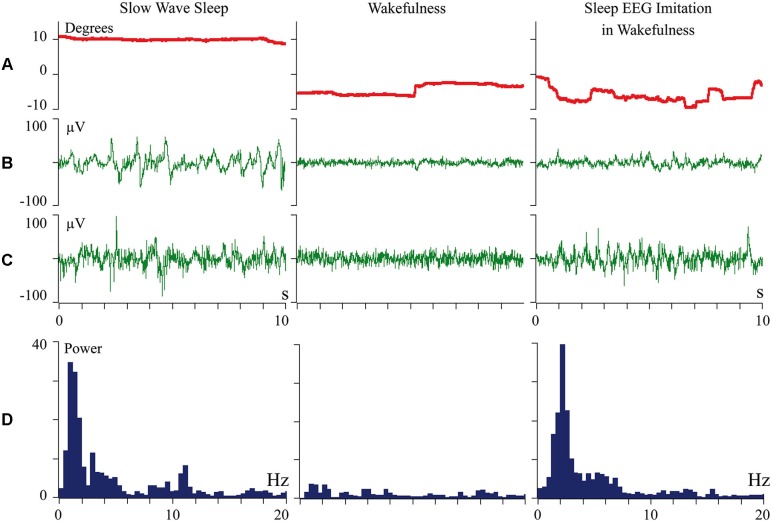
**Periodic optimal somatic stimulation during wakefulness evokes sleep like slow wave activity in the cat somatosensory cortex**. The rows: **(A)** vertical component of eye movements which helps to distinguish sleep (upward deviation) from wakefulness (downward deviation); **(B)** general EEG recorded between electrodes over temporal and frontal cortical areas of the cat; **(C)** local field potentials (local EEG) recorded between two tungsten microelectrodes located 300 mm one from another within the cortical somatosensory area 5; **(D)** power spectrum of the local EEG presented above in **panel C**. All parameters were collected during SWS (left column), during passive wakefulness (middle column), and during the procedure of “sleep EEG imitation” by visual stimulation in wakefulness, which produced strong excitation of the cortical neurons recorded by the microelectrodes (right column). Technical details of the study in Pigarev et al. (2013).

We proposed that difference of cortical afferentation in wakefulness and sleep might define the observed difference of neuronal activity in SWS and wakefulness. To check this proposal, general EEG and eye movements were recorded in behaviorally awake cats during SWS and active wakefulness between electrodes located over temporal and frontal cortical areas. In addition, we recorded neuronal activity and local field potentials (local EEG) from visual (Figure 1) and somatosensory (Figure 2) cortical areas using bipolar tungsten microelectrodes with distances of about 300 μm between the tips of the electrodes. During SWS the animal eyes were always deviated upward, and this allowed us to easily distinguish periods of sleep from active wakefulness in the obtained recordings. For every group of recorded neurons we applied the optimal parameters of stimulation (either visual or somatosensory), and delivered these stimuli in a rhythmic manner. We called this procedure “sleep EEG imitation in wakefulness”. Using this procedure (right column), in actively awake cats, we got burst neuronal firing (not shown) and EEG slow waves, which were indistinguishable from, or even higher than, those which we had observed during the periods of natural SWS (left column). These sleep-like waves were especially well visible in the channel of the local field potentials (Figures 1C and 2C, right columns) because the local EEG reflected activity of the neurons for which we used the optimal stimulation. The general EEG reflected averaged activity collected from the large cortical territory, including those neurons for which applied stimuli were not optimal. Nevertheless, some sleep-like waves were seen even in the general EEG (Figures 1B and 2B, right columns). In row D in Figures 1 and 2 we present power spectrums calculated for the 10 s fragments of the local EEG shown in row C of the corresponding column. It is seen that spectral compositions of the local EEG for SWS (left column) and imitation of sleep EEG in wakefulness (right column) were rather similar, and both differed from the usual spectrum of quiet wakefulness (central column).

The presented observations supported an idea that patterns of the cortical afferentation, rather than the state of vigilance, determine the pattern of cortical activity. All above-mentioned considerations inclined us to conclude that switching to analysis of another flow of incoming information would be able to better explain the phenomenology of transition from wakefulness to sleep. The temporal organization of these incoming signals, specific for the state of sleep, will define the pattern of cortical activity during sleep.

## Which signals could provide periodic and synchronous afferentation during sleep?

Animal physiology offered the answer on this question; that it can be periodic activity of various visceral systems, e.g., gastro-intestinal peristalsis, heart and respiratory activities. We proposed that during sleep the same brain neurons that in wakefulness process exteroceptive information of various modalities switch over to the analysis of interoceptive information coming from visceral systems. Rhythmic activities of different visceral systems define this periodic afferent flow towards the cortical areas, which is reflected in cortical SWS activity. Thus, the central nervous system during sleep might be involved in the process of visceral regulation (Pigarev, 2014).

According to this proposal, periods of local sleep are not the periods when “tired” brain areas stop processing of exteroceptive information in favor of self-recuperation. During periods of local sleep, normally working brain areas respond to warning messages from the internal organs and switch to the processing of the alarming visceral afferentation. Within the frame of this hypothesis, we should “think differently” about the nature of sleep and local sleep.

This suggestion may be too fantastic for the brain paradigm generally accepted at present. This paradigm was established mainly on the basis of data collected for the state of wakefulness. On the other hand, our “fantastic” proposal opened the way for its experimental validation in simple experiments, which could not be conducted without this theoretical background. Below, we offer a short review of the experiments performed to investigate such nontrivial predictions of the visceral hypothesis of sleep.

## Experimental validation of the visceral hypothesis of sleep

First of all, to check this hypothesis responses of different cortical regions to extero- and interoceptive stimulation during sleep and wakefulness were compared. These experiments were started from the visual cortical areas. Visual areas were selected for these experiments because they were well studied, and it was generally recognized that in behaviorally awake animals neurons of these areas were responding exclusively to visual stimulation. In addition, one of us (Ivan N. Pigarev) had considerable experience in investigation of various visual areas in behaviorally awake animals. Later, similar experiments were conducted with neurons not only in occipital, but also in frontal and parietal cortical areas.

**Figure 3:**
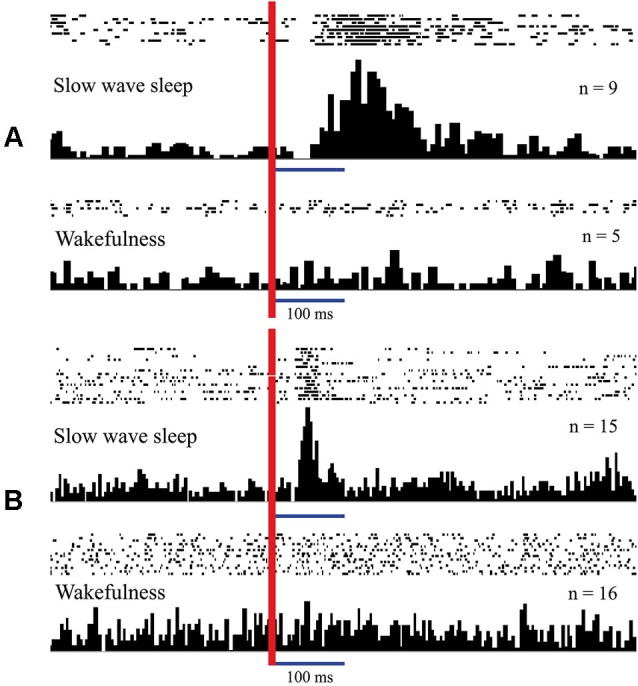
**Responses of neurons in the cat cortical visual area V1 (A) and somatosensory area 5 (B) to intraperitoneal electrical stimulation delivered in sleep and in wakefulness.** Responses presented as rasters where every line corresponds to single stimulation trial. Dots represent single spikes. Below are averaged histograms. Vertical red line–moment of intraperitoneal stimulation. n—number of averaged trials. Technical details of the study in Pigarev (1994).

In Figure 3 (adopted from Pigarev, 1994; Pigarev and Pigareva, 2012) we show responses of complex neurons in visual cortical area V1 (**panel A**) and somatosensory area 5 (**panel B**) of cats to electrical intraperitoneal stimulation delivered in SWS and in wakefulness. These neurons in the state of wakefulness responded to visual and somatosensory stimuli respectively. During SWS both neurons responded to electrical stimulation of the area of the small intestine, and these apparent responses immediately disappeared in REM sleep and after awakening.

Similar experiments were conducted with monkeys, where evoked responses to intraperitoneal electrical stimulation were recorded above the cortical visual area V1 (Pigarev et al., 2006). Evoked responses were again obtained only during SWS, and disappeared during REM sleep and in the state of wakefulness.

**Figure 4:**
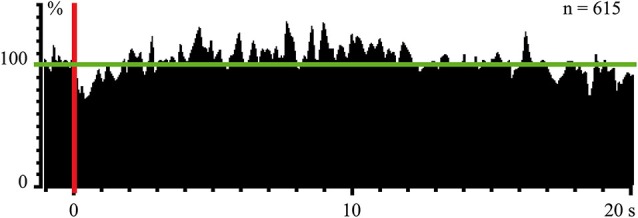
**Population response of 61 neurons (615 trials) in monkey’s cortical visual area V4 to magnetic stimulation of stomach during SWS.** Vertical red line–moment of stimulation. Horizontal green line indicate mean background firing rate before stimulation taken as 100%. Technical details of the study in Pigarev et al. (2008).

In experiments with one monkey we used magnetic stimulation, with the coil located close to the surface of the monkey’s stomach. In response to magnetic pulses, which did not wake the animal, we obtained cortical evoked responses, recorded by the electrodes above the occipital pole of the skull. These responses were observed again only during SWS (Pigarev et al., 2008). Simultaneous recording of the neuronal activity in the visual area V4 revealed a strong short latency inhibition in response to these magnetic pulses, which was obviously visible even in the population response of 61 neurons (Figure 4). After this short latency inhibition, the delayed (5–15 s) activation of the background firing took place. This result deserves attention because receptive fields in area V4 had small excitatory areas and huge inhibitory surrounds. The applied magnetic pulses could activate those parts of visceral organs, which projected to this huge inhibitory periphery of the studied receptive fields. On the other hand, after some delay, peristaltic waves provoked by the stimulation could reach regions, which projected to the central excitatory part of the receptive fields, causing the observed delayed activation. All these responses to magnetic stimulation again disappeared in wakefulness.

In experiments with rabbits (Pigarev et al., 2004), we also recorded evoked responses to electrical intraperitoneal stimulation in visual and somatosensory cortical areas, which appeared again exclusively during SWS.

It has been argued that electrical and magnetic stimuli are not natural, and that observed effects could have a non-specific origin. Although the main information concerning organization of the nervous system was obtained using electrical stimulation, it would be much more important to demonstrate a functional link between visceral organs and cortical areas during sleep in natural conditions, without any artificial stimulation.

Such experiments were conducted with the help of our colleagues from the Pavlov Institute of Physiology (St. Petersburg), prof. V. A. Bagaev and I. I. Busigina. Recording electrodes were implanted in the walls of the small intestine and stomach of cats, together with stomach fistula. With this approach, in addition to cortical neuronal activity, EEG, and ocular movements we could record myoelectrical activity of small intestine and stomach, and to change intragastric contents.

**Figure 5:**
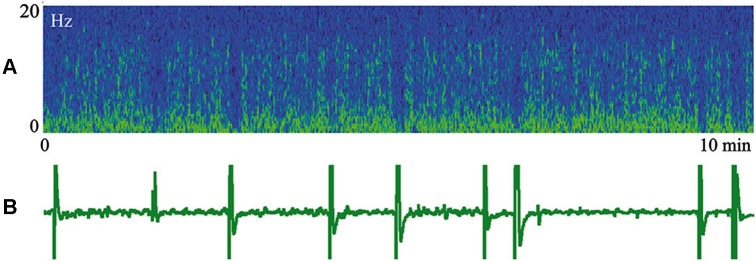
**Episode of SWS**. **(A)** Spectrogram of the cat cortical EEG. Yellow color indicates higher power. **(B)** Myoelectrical activity of stomach. Technical details of the study in Pigarev et al. (2013).

In Figure 5 we present a spectrogram of cat cortical EEG (A) recorded simultaneously with myoelectrical activity of the stomach (B) during an episode of SWS. In the spectrogram, yellow colors indicate higher power, and periodic vertical blue fragments indicate moments of short desynchronizations connected with lack of low frequency components. These desynchronized intervals are well known to anybody who has recorded EEG during SWS. It was previously demonstrated (Oniani et al., 1974) that, behaviorally, sleep was not interrupted during these periods, and thresholds for awakening during such short desynchronizations still were very high. What was new in the presented figure was a surprising coincidence of these EEG desynchronizations with the appearance of periodic migrating myoelectrical complexes in the stomach (short vertical inclinations in B). There was no need for any special analysis in order to notice such coincidence. Simultaneous appearance of the migrating myoelectrical complexes in stomach activity and short desynchronizations in the cortical EEG usually happened during intervals of 10–20 min of SWS. The observed coincidence of these effects can disappear for a while and appear again later. This was a very robust effect, observed in most of our sleep recordings, which included the periods of corresponding stomach activity.

More impressive were results of those experiments where we have studied interaction of the neuronal activity in various cortical visual areas and myoelectrical activity from the wall of the duodenum (Pigarev et al., 2013). It was demonstrated that about one third of more than 200 of the studied cortical neurons during SWS established a causal relationship with the activity of the duodenum during SWS. Even more, these neurons demonstrated selectivity to particular types of duodenal rhythmicity. Some neurons preferred simple duodenal waves, and others responded only to waves with spike potentials. Such a relationship was never observed in wakefulness.

Finally it was found that changes of the intragastric medium (water infusion via fistula into the stomach) performed in the period of SWS lead to changes in the EEG pattern and temporal reorganization of the background neuronal spiking, revealed by Fano factor analysis (Pigarev et al., 2010).

**Figure 6:**
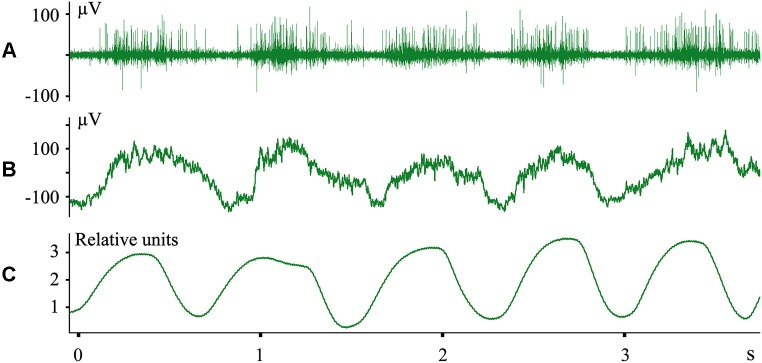
**Neuronal activity in the cat primary visual cortex synchronized with respiration during SWS. (A)** Multiunit activity; **(B)** local field potentials (local EEG); **(C)** nasal air flow in relative units. Technical details of the study in Pigarev et al. (2013).

We do not imply that only the structures of the digestive system are represented in the cerebral cortex during sleep. In other experiments we recorded evoked responses to heartbeats during sleep (Pigarev and Feodorov, 2012). An example of neuronal firing and local field potentials in the visual area V1, which synchronized with respiration during SWS, is shown in Figure 6. Dr. M. Lebedev, during experiments with monkeys under anesthesia, also observed unexpected neuronal activity synchronized with respiration in somatosensory cortical areas within the representation of the hind paw (personal communication).

## The visceral sleep theory and observations of “slow wave” activity in the cortical slabs and slices

According to the visceral sleep theory, patterns of periodic activation coming from the visceral organs determine the oscillating picture of cortical activity during SWS. The desynchronized pattern of cortical EEG during REM sleep can be connected with afferentation coming to the cerebral cortex from visceral systems lacking obvious rhythmic activity, e.g., liver, kidneys, reproductive organs and, finally, the brain itself. The brain’s status within this theory is obviously dual. On the one hand, the brain is the central processor which controls behavior in the environment during wakefulness and defines recovery of all visceral organs during sleep. However, on the other hand, the brain itself is an enormously complicated visceral organ, which certainly should be in need of service. How and when such brain self-service is realized is a challenging question. It may happen, for example, during particular phases of REM or SWS, or it may be organized as a permanent service, e.g., by glial cells. The recently discovered “glymphatic” mechanism may reflect elements of such brain self-servicing (Nedergaard, 2013; Xie et al., 2013). Various other options can be offered, but that is a topic for future studies.

Our approach to sleep function supposes that cortical activity during sleep is defined by the afferent flow coming to the cerebral cortex from various visceral organs. On the other hand, there is substantial evidence that sleep-like activity can be generated in cortical slabs (Timofeev et al., 2000) and isolated cortical slices (Sanchez-Vives and McCormick, 2000) without any interoceptive inputs. However, we do not think that these observations are inconsistent with our theoretical proposal. Of relevance is the important discovery of Steriade et al. (2001), who performed the first intracellular recordings of neuronal cortical activity in naturally sleeping cats. They found that waves of hyperpolarization reflected as periodic silent pauses in neuronal firing during SWS were connected not with active inhibition, but with disfacilitation caused by the lack of excitatory inputs to these cortical neurons. At the time of their study it was generally recognized that, during sleep, the cerebral cortex was disconnected from any afferent inputs, and they had to conclude that, “during SWS, neocortical neurons may be engaged in information processing of internally generated signals‥.”, which provided such excitatory inputs. As a source of such “internally generated signals” they considered intracortical excitation (Steriade et al., 2001).

The studies performed on the isolated cortical slices demonstrated that, in certain conditions, it was possible to evoke periodic neuronal discharges, which had some features of similarity with real SWS oscillations. Later it was shown in experiments on thalamo-cortical slices that activation of the thalamo-cortical neurons dominates in triggering such cortical oscillations (Contreras and Steriade, 1995; Rigas and Castro-Alamancos, 2007). In a review (Crunelli and Hughes, 2010) it was recognized that most likely several mechanisms might elicit the cortical slow waves. We propose that “internally generated signals”, which define cortical waves during SWS, are actually coming from various visceral systems using the same thalamo-cortical pathways, which activate cortical neurons during wakefulness.

## The pathways for the visceral afferentation to the cerebral cortex during sleep

One may inquire about the ways by which the information from various visceral systems may reach “the same thalamo-cortical pathways”. For the somatosensory system it is well investigated. It is known since early anatomical studies in the 19th century (Head, 1896), that visceral and somatosensory afferents terminate at the same neurons in the spinal cord, and thus visceral information may travel to the cerebral cortex through the fibers of the somatosensory columns. The fact of such combined projections was confirmed in many studies (e.g., Kuo et al., 1981; Cervero, 1983; Cervero et al., 1984; Akeyson and Schramm, 1994; Perry and Lawson, 1998) and this overlap is regarded as the most probable mechanism of the referred pains (Head, 1896; Arendt-Nielsen and Svensson, 2001; Peles et al., 2004; Hobson et al., 2010).

However, this overlap created a yet unresolved problem–how the central nervous system manages to distinguish spikes coming by a single fiber from so different sources. Our hypothesis offers a solution to this problem. The transmission of the somatosensory information happens during wakefulness, while visceral information is transmitted to the central nervous system during sleep, when muscles are relaxed and movements are excluded.

To our knowledge, pathways of the visceral information to the visual cortical areas have never been investigated. Here we offer only some considerations. It is well known that, in the main thalamic visual relay (lateral geniculate nucleus), retinal synaptic terminals form only one third of all synaptic terminals. Another third of terminals belong to backward cortico-thalamic projections. The remaining one third of terminals is of non-visual origin, and come from the pontine and the brain stem regions (Hughes and Mullikin, 1984). Activation from pontine nuclei reaches the lateral geniculate nucleus during sleep, especially during REM sleep, and reflects in the visual cortical areas as well known ponto-geniculo-occipital waves (Brooks and Bizzi, 1963). The origin of this pontine activity has not yet been investigated. On the other hand, pontine and brain stem projections to the lateral geniculate nucleus come from the regions of entering and passage of various visceral nerves. Therefore, their link with visceral information is very probable.

## K-complexes and visceral afferentation, use dependency and sleep homeostasis

In discussions of cortical visceral activation during sleep, one often argues that these visceral responses can be of nonspecific origin, resembling sensory-triggered K-complexes. This topic was investigated in detail in our special study (Pigarev et al., 2011). It was shown that visually induced K-complexes had absolutely specific origin. Even more, K-complexes could be induced by sensory stimuli only during short intervals of developing sleep. In contrast, cortical visceral responses could be recorded during periods of deep SWS, when K-complexes could not be induced by any sensory stimulation. In addition, we presented arguments indicating that visually induced K-complexes were also of visceral origin.

The attempts to link various visceral events with the elements of EEG recorded during sleep, e.g., K-complexes, has been undertaken in many studies (Pampiglione and Ackner, 1958; Johnson and Karpan, 1968; Halász et al., 1985; Heald et al., 1989; Hornyak et al., 1991; Okada et al., 1991; Niiyama et al., 1996; Monstad and Guilleminault, 1999; Tank et al., 2003). The same idea was expressed in a study of Cash et al. (2009), where it was suggested that spontaneous K-complexes appearing in the EEG during transition from wakefulness to sleep could be induced by a “sensory stimulus occult to the investigator (e.g., gastric)”.

In the middle of the previous century, cortical responses to the stimulation of various visceral nerves were described and intensively investigated in several laboratories (e.g., Bailey and Bremer, 1938; Amassian, 1951; Patton and Amassian, 1952; Gardner et al., 1955; Chernigovskiy, 1960). These studies were performed in acute experiments under anesthesia. However, in later experiments without anesthesia, these results could not be reproduced. In wakefulness, neurons in these areas responded only to visual or somatosensory stimulation. Thus, cortical responses to visceral stimulation were regarded as probable artifacts of anesthesia.

In our studies we have demonstrated that without any anesthesia, in natural conditions, cortical areas do establish connections with visceral organs, but this link is functionally active only during sleep. Involvement of the highest levels of the central nervous system, up to the cerebral cortex in mammals, in the processing of visceral information during periods of sleep may be the main, if not the exclusive, function of sleep.

Here we should come back to the above-mentioned effect of “use dependency”, which is widely explored now as an experimental argument in favor of the concepts that sleep is necessary for brain recovery (Kattler et al., 1994; Rector et al., 2005; Huber et al., 2006). This effect can also be explained by the visceral sleep theory. High activation of some cortical areas by intensive exteroceptive stimulation during wakefulness will lower the neuronal thresholds of the neurons in these areas (e.g., due to the LTP mechanism). As a result, during sleep, these neurons will respond more strongly to visceral stimulation, coming to the same neurons through the same synaptic connections. Consequently the δ-power of the EEG will grow.

Discussing the visceral sleep theory we would like to draw attention to the distinguished theory of A. Borbély—the two-process model of sleep regulation (Borbély, 1982; Borbély and Achermann, 1999). This model proposes that sleep is regulated by the interaction of two processes—homeostatic and circadian. The homeostatic process allows for a constant amount of sleep during 24 h. Visceral sleep theory offers the physiological framework of this homeostatic mechanism. Homeostasis of all visceral systems is supported during sleep due to the involvement of all the cerebral cortex in the processing of information from internal organs. These informational processes define the total length of sleep.

## Mechanism of sleep initiation and features of local sleep

The visceral sleep theory explains the logic behind the initiation of sleep. Indeed, a mismatch between the current parameters of any visceral system and the genetically determined range for these parameters would provide the feeling of tiredness, or sleep pressure. If an environmental situation allows sleep, an organism would transit to normal total sleep in all cortical areas. Actually the chain of events for sleep initiation is more complicated. Evaluation of the visceral problems may need engagement of the mechanisms of emotions and such structures as hippocampus, amygdala and prefrontal cortex. All those questions were discussed in our recent article (Pigarev and Pigareva, 2013), and we will not continue this topic here. Now we would like to go back to the phenomenon of local sleep and to discuss it within the frame of the presented visceral sleep theory.

In the cases when, because of visceral problems, the need for sleep is dramatically increased, but environmental conditions do not allow sleep to occur, sleep may progress only in some cortical areas in still behaviorally active organisms. According to the information cited above, the development of sleep starts from the most recent, “high order” cortical areas. The proportion of such areas is highest in the frontal pole of the brain. This might underlie the reported fronto-occipital trend in the development of sleep.

It is logical to propose that those behavioral tasks, which do not need engagement of the highest cortical resources would be normally realized even in conditions when part of the brain is sleeping. However, in the situation when all cortical computational ability is required for decision making in a complicated problem, local sleep may lead to severe and often dramatic behavioral errors.

Conditions of local sleep development indicate that local sleep is very probable when it is necessary to remain awake during periods of high (natural) sleep pressure. For humans this might happen during work at the time of the maximal sleepiness. For rats, local sleep can accompany experiments during the light phase of the day, if the rats are caged in animal houses with non-inverted conditions of illumination.

People with habitual or forced short length of night sleep are at permanent risk of partial sleep development. For some professions partial sleep provoked by such chronic sleep deprivation may not cause any troubles. However, for professions connected with responsible and complex decision making, especially during night shifts, the dangerous consequences of local sleep are very high. For these professions, any visceral disorders, especially in the gastro-intestinal system, may dramatically increase the risk of wrong decisions.

Besides the negative effects of partial sleep for mental ability due to the disengagement of some cortical areas from the intellectual work, one should also not neglect the possible negative consequences of this phenomenon for the visceral health of an organism. As participation of all cortical areas in information processing is essential for the efficient solution of complex problems in wakefulness, for efficient management of visceral systems, all cortical areas should be involved into the processing of visceral information. Appearance of local sleep during wakefulness means that the length of total sleep during the nighttime was not sufficient, and may indicate hidden problems in some visceral systems.

## Conclusion

The detailed investigation of brain involvement in the regulation of the various visceral systems during sleep is a goal for further studies. At present, following the visceral theory of sleep, we can state that efficient and sufficient sleep, together with visceral health, might be the cheapest, safest and most pleasant way to augment brain function.

## Author Contributions

Both co-authors have equal contribution to all steps of preparation of this article and both approved the version to be published.

## Conflict of interest statement

The authors declare that the research was conducted in the absence of any commercial or financial relationships that could be construed as a potential conflict of interest.
